# Supplementary material for: Bioassay-Guided Isolation of Anti-Inflammatory Constituents of the Subaerial Parts of Cyperus articulatus (Cyperaceae)
Source: Molecules. 2022 Sep 13;27(18):5937. doi: 10.3390/molecules27185937 (PMC9504922; doi:10.3390/molecules27185937)
Supplement: Supplementary file 1 [file molecules-27-05937-s001.zip › molecules-1897780-supplementary.pdf]

Supplementary information

Bioassay-guided isolation of anti-inflammatory constituents from the subaerial parts of *Cyperus articulatus* (Cyperaceae)

Domenic Mittas [1], Monizi Mawunu [2], Giorgia Magliocca [3], Thea Lautenschläger [4], Stefan Schwaiger [1,*], Hermann Stuppner [1], Stefania Marzocco [3]

1 Institute of Pharmacy/Pharmacognosy, Center for Molecular Biosciences Innsbruck, University of Innsbruck, Innrain 80/82, 6020 Innsbruck, Austria
2 University of Kimpa Vita, Province of Uíge, Rua Henrique Freitas No. 1, Bairro Popular, Uíge, Angola
3 Department of Pharmacy, University of Salerno, Via Giovanni Paolo II 132, I-84084 Fisciano SA, Italy
4 Department of Biology, Institute of Botany, Faculty of Science, Technische Universität Dresden, 01062 Dresden, Germany

* Corresponding author. Tel.: +43 51250758409. E-mail address: stefan.schwaiger@uibk.ac.at

INDEX

Spectroscopic data of compounds isolated 3

Phytochemical and pharmacological analysis of the most active fractions......................... 8

Figure S1. Cell viability of the obtained PE, DEE, PRC, EtOAc, 1-BuOH and H_2O fractions. 8
Figure S2. HPLC-UV chromatogram (205 nm) of DEE subfractions A–R after separation by *NP*-MPLC. 9
Figure S3. Cell viability of DEE subfractions A–R of the methanolic extract of the subaerial parts of *C. articulatus*. 10
Figure S4. GC chromatogram and peak assignment of the PE fraction of the methanolic extract of the subaerial parts of *C. articulatus*. 11
Table S1. Antiproliferative activity and NO production inhibition of compounds **1**–**11** obtained from the PE fraction of the subaerial parts of *C. articulatus*. 12
Table S2. Antiproliferative activity of compounds **12**–**19** obtained from the DEE fraction of the subaerial parts of *C. articulatus*. 13
Figure S5. Comparison of the chromatograms (205 nm) of the HPLC-UV analysis of extract subfractions of *S. holoschoenus* and *C. articulatus*.. 14

Spectroscopic data of compounds isolated

*Copa-3-en-2α-ol (**1**)* [$C_{15}H_{24}O$]: White needles; $[\alpha]_D^{20}$ - 24.2 ($CHCl_3$; *c* 0.05); ^{1}H NMR (400.19 MHz, $CDCl_3$) δ_H (in ppm) 5.36 (1H, dq, *J* = 3.1, 1.6 Hz, H-3), 4.32 (1H, brs, H-2), 2.19 (1H, ddd, *J* = 6.3, 3.5, 1.7 Hz, H-1), 1.84 (1H, brs, H-6), 1.76 (1H, m, H-9a), 1.72 (3H, t, *J* = 1.6 Hz, H-15), 1.63 (1H, m, H-8a), 1.61 (1H, m, H-7), 1.59 (1H, s, H-5), 1.59 (1H, m = H-9b), 1.53 (1H, m, H-8b), 1.52 (1H, m, H-11), 0.88 (3H, d, *J* = 3.2 Hz, H-13), 0.87 (3H, d, *J* = 3.2 Hz, H-12), 0.82 (3H, s, H-14). ^{13}C NMR (150.91 MHz, $CDCl_3$) δc (in ppm) 148.72 (C, C-4), 119.01 (CH, C-3), 70.74 (CH, C-2), 55.45 (CH, C-5), 48.80 (C, C-10), 44.60 (CH, C-7), 43.98 (CH, C-1), 41.73 (CH, C-6), 36.55 (CH_2, C-9), 32.23 (CH, C-11), 22.87 (CH_3, C-15), 21.88 (CH_2, C-8), 20.15 (CH_3, C-13), 19.66 (CH_3, C-12), 19.11 (CH_3, C-14). LC-ESI-MS (positive mode): *m/z* 203.1 $[M+H-H_2O]^+$ (100), 405.2 $[2M-2OH]^+$ (84), 243.1 $[M+Na]^+$ (6).

*Caryophyllene oxide (**2**)* [$C_{15}H_{24}O$]: Colorless oil; $[\alpha]_D^{20}$ - 26.0 ($CHCl_3$; *c* 0.05); ^{1}H NMR (400.19 MHz, $CDCl_3$) δ_H (in ppm) 4.98 (1H, s, H-13a), 4.86 (1H, s, H-13b), 2.88 (1H, dd, *J* = 10.6, 4.3 Hz, H-5), 2.62 (1H, dd, *J* = 19.4, 8.9 Hz, H-9), 2.34 (1H, ddd, *J* = 12.8, 8.2, 4.4 Hz, H-7a), 2.25 (1H, ddt, *J* = 12.4, 8.5, 4.3 Hz, H-6a), 2.12 (1H, m, H-7b), 2.09 (1H, dt, *J* = 12.3, 3.6 Hz, H-3a), 1.76 (1H, t, *J* = 9.8, H-1), 1.69 (1H, dd, *J* = 10.6, 8.3 Hz, H-10a), 1.64 (1H, m, H-2a), 1.62 (1H, m, H-10b), 1.42 (1H, m, H-2b), 1.33 (1H, dddd, *J* = 12.8, 10.7, 8.1, 4.5 Hz, H-6b), 1.20 (3H, s, H-12), 1.01 (3H, s, H-15), 0.98 (3H, s, H-14), 0.96 (1H, dd, *J* = 13.2, 5.0 Hz, H-3b). ^{13}C NMR (150.91 MHz, $CDCl_3$) δc (in ppm) 152.01 (C, C-8), 112.91 (CH_2, C-13), 63.92 (CH, C-5), 60.00 (C, C-4), 50.89 (CH, C-1), 48.88 (CH, C-9), 39.90 (CH_2, C-10), 39.30 (CH_2, C-3), 34.17 (C, C-11), 30.34 (CH_2, C-6), 30.03 (CH_3, C-15), 29.93 (CH_2, C-7), 27.35 (CH_2, C-2), 21.76 (CH_3, C-14), 17.14 (CH_3, C-12). LC-ESI-MS (positive mode): *m/z* 221.2 $[M+H]^+$ (100), 441.3 $[2M+H]^+$ (6).

*Humulene epoxide-II (**3**)* [$C_{15}H_{24}O$]: Colorless oil; $[\alpha]_D^{20}$ - 23.2 ($CHCl_3$; *c* 0.1); ^{1}H NMR (600.19 MHz, $CDCl_3$) δ_H (in ppm) 5.28 (1H, ddd, *J* = 15.6, 10.1, 5.3 Hz, H-3), 5.15 (1H, d, *J* = 15.8 Hz, H-4), 5.00 (1H, dt, *J* = 9.2, 3.6 Hz, H-7), 2.57 (1H, dd, *J* = 12.4, 5.3 Hz, H-2a), 2.53 (1H, dd, *J* = 10.2, 3.9 Hz, H-11), 2.24 (1H, m, H-9a), 2.17 (1H, ddd, *J* = 16.7, 9.4, 4.8 Hz, H-10a), 2.11 (1H, dd, *J* = 12.2, 9.5, 5.2 Hz, H-9b), 1.99 (1H, dd, *J* = 13.7, 9.2 Hz, H-6a), 1.87 (1H, dd, *J* = 13.8, 5.9 Hz, H-6b), 1.64 (1H, dd, *J* = 12.5, 10.1 Hz, H-2b), 1.56 (3H, d, *J* = 4.2 Hz, H-15), 1.35 (1H, ddt, *J* = 13.2, 10.2, 5.1 Hz, H-10b), 1.30 (3H, s, H-12), 1.11 (3H, s, H-14), 1.08 (3H, s, H-13). ^{13}C NMR (150.91 MHz, $CDCl_3$) δc (in ppm) 143.28 (CH, C-4), 132.08 (C, C-8), 125.88 (CH, C-7), 122.25 (CH, C-3), 63.38 (C, C-1), 62.10 (CH, C-11), 42.73 (CH_2, C-2), 40.37 (CH_2, C-6), 36.77* (CH_2, C-9), 36.66* (C, C-5), 29.15 (CH_3, C-13), 25.61 (CH_3, C-14), 24.89 (CH_2, C-10), 17.34 (CH_3, C-12), 15.23 (CH_3, C-15). *The assignments of C-5 and C-9 may be exchangeable and are labeled with an asterisk. LC-ESI-MS (positive mode): *m/z* 221.2 $[M+H]^+$ (100), 441.2 $[2M+H]^+$ (13), 243.1 $[M+Na]^+$ (11).

*Mustakone (**4**)* [$C_{15}H_{22}O$]: Colorless oil; $[\alpha]_D^{20}$ - 13.2 (MeOH; *c* 0.1); ^{1}H NMR (400.19 MHz, $CDCl_3$) δ_H (in ppm) 5.74 (1H, q, *J* = 1.5 Hz, H-3), 2.68 (1H, dd, *J* = 6.9, 1.7 Hz, H-1), 2.67 (1H, d, *J* = 2.8 Hz, H-6), 2.00 (3H, d, *J* = 1.5 Hz, H-14), 1.98 (1H, dd, *J* = 6.7, 1.4 Hz, H-5), 1.88 (1H, m, H-9a), 1.76 (1H, m, H-8a), 1.72 (1H, m, H-9b), 1.63 (1H, s, H-7), 1.52 (2H, m, H-8b, H-11), 0.98 (3H, s, H-15), 0.86* (3H, d, *J* = 6.7 Hz, H-12), 0.85* (3H, d, *J* = 6.7 Hz, H-13). ^{13}C NMR (150.91 MHz, $CDCl_3$) δc (in ppm) 204.24 (C, C-2), 170.13 (C, C-4), 121.53 (CH, C-3), 57.44 (C, C-10), 56.68 (CH, C-5), 56.12 (CH, C-6), 54.65 (CH, C-1), 45.56 (CH, C-7), 36.84 (CH_2, C-9), 31.91 (CH, C-11), 23.82 (CH_3, C-14), 22.13 (CH_2, C-8), 20.44 (CH_3, C-15), 20.09 (CH_3, C-12), 19.66 (CH_3, C-13). *The assignments of H-12 and H-13 may

be exchangeable and are labeled with an asterisk. LC-ESI-MS (positive mode): *m/z* 219.2 $[M+H]^+$ (100), 437.2 $[2M+H]^+$ (100), 459.3 $[2M+Na]^+$ (46).

*Kobusone (**5**)* [$C_{14}H_{22}O_2$]: White solid; $[\alpha]_D^{20}$ - 95.0 ($CHCl_3$; *c* 0.1); ^{1}H NMR (600.19 MHz, $CDCl_3$) δ_H (in ppm) 3.06 (1H, m, H-9), 2.70 (1H, dd, *J* = 10.1, 5.0 Hz, H-5), 2.55 (2H, m, H-7), 2.40 (1H, m, H-6a), 2.15 (1H, dt, *J* = 13.2, 3.7 Hz, H-3a), 2.07 (1H, t, *J* = 10.3 Hz, H-10a), 1.94 (1H, ddd, *J* = 10.6, 8.8, 1.3 Hz, H-1), 1.66 (2H, m, H-2, H-10b), 1.53 (1H, m, H-2), 1.45 (1H, dddd, *J* = 13.2, 10.1, 6.2, 4.8 Hz, H-6b), 1.31 (3H, s, H-14), 1.04 (3H, s, H-12), 1.03 (3H, s, H-13), 0.95 (1H, td, *J* = 13.3, 4.4 Hz, H-3b). ^{13}C NMR (150.91 MHz, $CDCl_3$) δc (in ppm) 214.39 (C, C-8), 61.84 (CH, C-5), 59.16 (C, C-4), 52.80 (CH, C-9), 51.49 (CH, C-1), 39.17 (CH_2, C-3), 37.86 (CH_2, C-7), 35.45 (CH_2, C-10), 34.68 (C, C-11), 29.49 (CH_3, C-13), 26.64 (CH_2, C-2), 24.94 (CH_2, C-6), 22.37 (CH_3, C-12), 16.37 (CH_3, C-14). LC-ESI-MS (positive mode): *m/z* 223.0 $[M+H]^+$ (100), 245.1 $[M+Na]^+$ (10).

*Cyperotundone (**6**)* [$C_{15}H_{22}O$]: White solid; $[\alpha]_D^{20}$ + 30.0 (MeOH; *c* 0.1); ^{1}H NMR (400.19 MHz, $CDCl_3$) δ_H (in ppm) 2.59 (1H, dd, *J* = 18.9, 7.1 Hz, H-6a), 2.30 (1H, d, *J* = 19.0 Hz, H-6b), 2.17 (1H, m, H-10), 2.15 (1H, d, *J* = 17.4 Hz, H-2a), 2.01 (1H, d, *J* = 17.4 Hz, H-2b), 1.95 (2H, m, H-7, H-8a), 1.72 (3H, t, *J* = 1.4 Hz, H-14), 1.59 (1H, d, *J* = 13.6 Hz, H-9a), 1.41 (1H, dt, *J* = 13.0, 6.7, 3.5 Hz, H-8b), 1.10 (3H, s, H-12), 1.02 (1H, ddd, *J* = 12.5, 7.0, 2.0 Hz, H-9b), 0.74 (3H, s, H-13), 0.61 (3H, d, *J* = 6.4 Hz, H-15). ^{13}C NMR (150.91 MHz, $CDCl_3$) δc (in ppm) 211.18 (C, C-3), 181.91 (C, C-5), 133.54 (C, C-4), 58.85 (C, C-1), 45.51 (CH, C-7), 41.77 (C, C-11), 41.30 (CH_2, C-2), 33.88 (CH, C-10), 30.64 (CH_2, C-6), 28.52 (CH_2, C-9), 27.16 (CH_2, C-8), 24.97 (CH_3, C-13), 19.71 (CH_3, C-12), 16.97 (CH_3, C-15), 8.47 (CH_3, C-14). LC-ESI-MS (positive mode): *m/z* 219.1 $[M+H]^+$ (100), 459.3 $[2M+Na]^+$ (38).

*Humulene dioxide (**7**)* [$C_{15}H_{24}O_2$]: Colorless needles; $[\alpha]_D^{20}$ - 25.8 ($CHCl_3$; *c* 0.1); ^{1}H NMR (600.19 MHz, $CDCl_3$) δ_H (in ppm) 5.49 (1H, ddd, *J* = 15.6, 10.6, 5.0 Hz, H-3), 5.31 (1H, d, *J* = 16.5 Hz, H-4), 2.73 (1H, dd, *J* = 10.1, 5.0 Hz, H-11), 2.64 (1H, dd, *J* = 12.2, 5.0 Hz, H-2a), 2.48 (1H, d, *J* = 9.6 Hz, H-7), 2.20 (1H, tt, *J* = 13.8, 5.3 Hz, H-10a), 2.13 (1H, ddd, *J* = 13.6, 5.8, 2.4 Hz, H-9a), 1.65 (1H, t, *J* = 11.5 Hz, H-2b), 1.61 (1H, d, *J* = 14.3 Hz, H-6a), 1.37 (2H, m, H-6b, H-10b), 1.31 (6H, s, H-12, H-15), 1.20 (3H, s, H-14), 1.08 (3H, s, H-13), 1.08 (1H, m, H-9b). ^{13}C NMR (150.91 MHz, $CDCl_3$) δc (in ppm) 143.03 (CH, C-4), 122.72 (CH, C-3), 64.81 (CH, C-7), 63.51 (C, C-1), 60.46 (CH, C-11), 60.23 (C, C-8), 43.45 (CH_2, C-2), 38.50 (CH_2, C-6), 35.79 (C, C-5), 34.99 (CH_2, C-9), 30.82 (CH_3, C-13), 25.33 (CH_2, C-10), 23.50 (CH_3, C-14), 16.60* (CH_3, C-12), 16.56* (CH_3, C-15). *The assignments of C-12 and C-15 may be exchangeable and are labeled with an asterisk. LC-ESI-MS (positive mode): *m/z* 237.2 $[M+H]^+$ (100), 259.1 $[M+Na]^+$ (76), 275.0 $[M+K]^+$ (9); LC-ESI-MS (negative mode): *m/z* 235.7 $[M-H]^-$ (100).

*Humulene dioxide (**8**)* [$C_{15}H_{24}O_2$]: Colorless needles; $[\alpha]_D^{20}$ - 13.8 ($CHCl_3$; *c* 0.1); ^{1}H NMR (600.19 MHz, $CDCl_3$) δ_H (in ppm) 5.56 (1H, d, *J* = 15.9 Hz, H-4), 5.53 (1H, dd, *J* = 8.0, 4.3 Hz, H-3), 2.69 (1H, d, *J* = 10.9 Hz, H-7), 2.63 (1H, d, *J* = 9,2 Hz, H-11), 2.61 (1H, dd, *J* = 12.8, 3.3 Hz, H-2a), 2.23 (1H, dt, *J* = 13.9, 3.8 Hz, H-9a), 1.99 (1H, dt, *J* = 14.7, 4.1 Hz, H-10a), 1.81 (1H, dd, *J* = 13.5, 7.9 Hz, H-2b), 1.72 (1H, m, H-6a), 1.46 (2H, m, H-6b, H-10b), 1.39 (3H, s, H-12), 1.29 (1H, m, H-9b), 1.20 (3H, s, H-14), 1.18 (3H, s, H-15), 1.10 (3H, s, H-13). ^{13}C NMR (150.91 MHz, $CDCl_3$) δc (in ppm) 142.59 (CH, C-4), 123.94 (CH, C-3), 64.15 (C, C-1), 63.16 (2CH, C-7, C-11), 60.76 (C, C-8), 42.49 (CH_2, C-6), 40.83 (CH_2, C-2), 36.93 (CH_2, C-9), 34.68 (C, C-5), 30.25 (CH_3, C-13), 26.95 (CH_3, C-14), 23.68 (CH_2, C-10), 19.14

(CH_3, C-12), 16.47 (CH_3, C-15). LC-ESI-MS (positive mode): *m/z* 237.2 $[M+H]^+$ (100), 259.1 $[M+Na]^+$ (46); LC-ESI-MS (negative mode): *m/z* 235.9 $[M-H]^-$ (100).

*(-)-Guaia-1(10),11-dien-9-one (**9**)* [$C_{15}H_{22}O$]: Colorless needles; $[\alpha]_D^{20}$ - 49.0 ($CHCl_3$; *c* 0.1); ^1H NMR (600.19 MHz, $CDCl_3$) δ_H (in ppm) 4.73 (2H, d, *J* = 8.9 Hz, H-12), 2.83 (1H, m, H-5), 2.72 (1H, dd, *J* = 13.6, 12.2 Hz, H-8a), 2.57 (1H, m, H-2a), 2.53 (1H, dd, 13.9, 3.3 Hz, H-8b), 2.49 (1H, dd, *J* = 8.0, 4.3 Hz, H-7), 2.39 (1H, dt, *J* = 18.6, 7.2 Hz, H-2b), 2.28 (1H, p, *J* = 6.7 Hz, H-4), 1.82 (1H, m, H-3a), 1.77 (3H, d, *J* = 1.7 Hz, H-14), 1.75 (3H, s, H-13), 1.72 (1H, m, H-6a), 1.52 (2H, dddd, *J* = 13.9, 12.4, 7.2, 5.0 Hz, H-6b, H-3b), 0.89 (3H, d, *J* = 7.1 Hz, H-15). ^{13}C NMR (150.91 MHz, $CDCl_3$) δc (in ppm) 204.53 (C, C-9), 163.22 (C, C-1), 148.43 (C, C-11), 131.39 (C, C-10), 109.72 (CH_2, C-12), 46.76 (CH_2, C-8), 45.06 (CH, C-5), 39.66 (CH, C-7), 38.82 (CH, C-4), 32.57 (CH_2, C-2), 31.71 (CH_2, C-3), 31.47 (CH_2, C-6), 21.20 (CH_3, C-13), 14.83 (CH_3, C-14), 14.54 (CH_3, C-15). LC-ESI-MS (positive mode): *m/z* 219.0 $[M+H]^+$ (100), 459.2 $[M+Na]^+$ (47).

*Muurolane-2β,9β-diol-3-ene (**10**)* [$C_{15}H_{26}O_2$]: White powder; $[\alpha]_D^{20}$ - 73.0 ($CHCl_3$; *c* 0.1); ^1H NMR (400.19 MHz, $CDCl_3$) δ_H (in ppm) 5.76 (1H, dd, *J* = 5.6, 1.5 Hz, H-5), 3.95 (1H, brs, H-3), 2.32 (1H, brs, H-6), 1.94 (1H, pd, *J* = 6.9, 3.1 Hz, H-11), 1.80 (3H, brs, H-15), 1.74 (3H, m, H-1, H-2), 1.56 (1H, m, H-9a), 1.39 (3H, m, H-8, H-9b), 1.22 (3H, s, H-14), 1.19 (1H, m, H-7), 0.89 (3H, d, *J* = 6.9 Hz, H-12), 0.85 (3H, d, *J* = 6.9 Hz, H-13). ^{13}C NMR (150.91 MHz, $CDCl_3$) δc (in ppm) 134.21 (C, C-4), 129.86 (CH, C-5), 72.18 (C, C-10), 68.62 (CH, C-3), 42.72 (CH, C-7), 40.37 (CH, C-1), 34.90* (CH, C-6), 34.83* (CH_2, C-9), 30.69 (CH_2, C-2), 29.45 (CH_3, C-14), 27.21 (CH, C-11), 21.67 (CH_3, C-12), 21.18 (CH_3, C-15), 19.28 (CH_2, C-8), 15.60 (CH_3, C-13). *The assignments of C-6 and C-9 may be exchangeable and are labeled with an asterisk. LC-ESI-MS (positive mode): *m/z* 239.1 $[M+H]^+$ (100), 261.1 $[M+Na]^+$ (37), 499.2 $[2M+Na]^+$ (51); LC-ESI-MS (negative mode): *m/z* 237.1 $[M-H]^-$ (100).

*Corymbolone (**11**)* [$C_{15}H_{24}O_2$]: White powder; $[\alpha]_D^{20}$ + 18.6 ($CHCl_3$; *c* 0.1); ^1H NMR (400.19 MHz, $CDCl_3$) δ_H (in ppm) 4.74 (2H, s, H-12), 2.66 (1H, ddd, *J* = 16.2, 10.0, 3.6 Hz, H-2a), 2.42 (1H, m, H-2b), 2.39 (1H, m, H-6a), 2.32 (1H, tt, *J* = 12.6, 3.5 Hz, H-7), 1.89 (2H, m, H-3a, H-9a), 1.86 (1H, m, H-4), 1.75 (3H, s, H-13), 1.68 (2H, m, H-6b, H-8a), 1.60 (1H, m, H-3b), 1.43 (1H, dd, *J* = 13.8, 3.8, 2.1 Hz, H-9b), 1.37 (1H, ddd, *J* = 13.7, 4.0, 1.2 Hz, H-8b), 1.24 (3H, d, *J* = 0.8 Hz, H-14), 1.19 (3H, d, *J* = 7.5 Hz, H-15). ^{13}C NMR (150.91 MHz, $CDCl_3$) δc (in ppm) 215.96 (C, C-1), 149.69 (C, C-11), 109.04 (CH_2, C-12), 78.75 (C, C-5), 51.38 (C, C-10), 40.73 (CH, C-4), 39.50 (CH, C-7), 37.38 (CH_2, C-9), 34.39 (CH_2, C-2), 30.33 (CH_2, C-3), 28.17 (CH_2, C-6), 25.52 (CH_2, C-8), 21.26 (CH_3, C-13), 20.52 (CH_3, C-14), 17.90 (CH_3, C-15). LC-ESI-MS (positive mode): *m/z* 237.2 $[M+H]^+$ (100); LC-ESI-MS (negative mode): *m/z* 235.8 $[M-H]^-$ (100).

*p-Hydroxybenzoic acid (**12**)* [$C_7H_6O_3$]: Yellowish-white solid; ^{1}H NMR (600.19 MHz, MeOH-d_4) δ_H (in ppm) 7.87 (2H, d, *J* = 8.7 Hz, H-2, H-6), 6.81 (2H, d, *J* = 8.7 Hz, H-3, H-5). ^{13}C NMR (150.91 MHz, MeOH-d_4) δc (in ppm) 170.84 (C, C-7), 163.07 (C, C-4), 132.92 (2CH, C-2, C-6), 123.66 (C, C-1), 115.93 (2CH, C-3, C-5). LC-ESI-MS (negative mode): *m/z* 137.0 $[M-H]^-$ (100), 275.1 $[2M-H]^-$ (30).

*trans-p-Hydroxycinnamic acid (**13**)* [$C_9H_8O_3$]: White solid; ^{1}H NMR (600.19 MHz, MeOH-d_4) δ_H (in ppm) 7.58 (1H, d, *J* = 15.9 Hz, H-7), 7.45 (2H, d, *J* = 8.6 Hz, H-2, H-6), 6.81 (2H, d, *J* = 8.8 Hz, H-3, H-5), 6.29 (1H, d, *J* = 15.9 Hz, H-8). ^{13}C NMR (150.91 MHz, MeOH-d_4) δc (in ppm) 171.51 (C, C-9), 161.06 (C, C-4), 146.13 (CH, C-7), 131.00 (2CH, C-2, C-6), 127.40 (C, C-1), 116.79 (2CH, C-3, C-5), 116.34 (CH, C-8). LC-ESI-MS (positive mode): *m/z* 165.1 $[M+H]^+$ (100); LC-ESI-MS (negative mode): *m/z* 163.0 $[M-H]^-$ (100).

*2R/2S Dihydroluteolin (**14**)* [$C_{15}H_{12}O_6$]: Yellowish-white solid; $[\alpha]_D^{20}$ - 4.2 (MeOH; *c* 0.1); ^{1}H NMR (600.19 MHz, MeOH-d_4) δ_H (in ppm) 6.95 (1H, d, *J* = 1.7 Hz, H-6'), 6.82 (2H, s, H-2', H-5'), 5.92 (1H, brs, H-8), 5.90 (1H, d, *J* = 2.1 Hz, H-6), 5.31 (1H, dd, *J* = 12.7, 3.1 Hz, H-2), 3.10 (1H, dd, *J* = 17.1, 12.7 Hz, H-3a), 2.73 (1H, dd, *J* = 17.1, 3.1 Hz, H-3b). ^{13}C NMR (150.91 MHz, MeOH-d_4) δc (in ppm) 197.66 (C, C-4), 168.78*[1] (C, C-7), 165.45 (C, C-5), 164.88*[1] (C, C-9), 146.92 (C, C-4'), 146.54 (C, C-3'), 131.85 (C, C-1'), 119.24 (CH, C-2'), 116.26 (CH, C-5'), 114.71 (CH, C-6'), 103.26 (C, C-10), 97.17*[2] (CH, C-6), 96.31*[2] (CH, C-8), 80.50 (CH, C-2), 44.12 (CH_2, C-3). *[1,2] The assignments of C-7 and C-9 and of C-6 and C-8 may be exchangeable and are labeled with an asterisk. LC-ESI-MS (negative mode): *m/z* 287.0 $[M-H]^-$ (100), 575.0 $[2M-H]^-$ (57).

*4S/4R-4-Hydroxy-1,10-seco-muurol-5-ene-1,10-dione (**15**)* [$C_{15}H_{24}O_3$]: Colorless oil; $[\alpha]_D^{20}$ + 0.9 (MeOH; *c* 0.1); ^{1}H NMR (400.19 MHz, MeOH-d_4) δ_H (in ppm) 6.48 (1H, s, H-5), 2.61 (1H, ddd, *J* = 17.0, 6.3, 5.3 Hz, H-2a), 2.46 (1H, ddd, *J* = 17.0, 9.2, 6.1 Hz, H-2b), 2.29 (2H, t, *J* = 7.4 Hz, H-9), 2.28 (1H, t, *J* = 7.4 Hz, H-7), 2.13 (3H, brs, H-14), 2.10 (2H, brs, H-3), 1.92 (1H, m, H-8a), 1.73 (1H, ddt, *J* = 13.2, 7.9, 6.6 Hz, H-11), 1.62 (1H, ddt, *J* = 13.9, 11.4, 7.0 Hz, H-8b), 1.43 (3H, s, H-15), 0.92 (3H, d, *J* = 6.7 Hz, H-12), 0.80 (3H, d, *J* = 6.7 Hz, H-13). ^{13}C NMR (150.91 MHz, MeOH-d_4) δc (in ppm) 211.80 (C, C-10), 200.92 (C, C-1), 153.01 (CH, C-5), 139.94 (C, C-6), 69.30 (C, C-4), 45.19 (CH, C-7), 42.38 (CH_2, C-9), 37.80 (CH_2, C-3), 36.24 (CH_2, C-2), 32.76 (CH, C-11), 29.90 (CH_3, C-14), 27.41 (CH_3, C-15), 25.97 (CH_2, C-8), 21.19 (CH_3, C-13), 20.79 (CH_3, C-12). LC-ESI-MS (positive mode): *m/z* 235.1 $[M+H-H_2O]^+$ (100); LC-ESI-MS (negative mode): *m/z* 297.2 $[M-H+FA]^-$ (100).

*Piceatannol (**16**)* [$C_{14}H_{12}O_4$]: White powder; ^{1}H NMR (400.19 MHz, MeOH-d_4) δ_H (in ppm) 6.97 (1H, d, *J* = 1.9 Hz, H-2), 6.89 (1H, d, *J* = 16.2 Hz, H-7), 6.83 (1H, dd, *J* = 8.2, 1.9 Hz, H-6), 6.74 (1H, d, *J* = 16.1 Hz, H-8), 6.73 (1H, d, *J* = 8.2 Hz, H-5), 6.43 (2H, d, *J* = 2.1 Hz, H-10, H-14), 6.15 (1H, t, *J* = 2.1 Hz, H-12). ^{13}C NMR (150.91 MHz, MeOH-d_4) δc (in ppm) 159.64 (2C, C-11, C-13), 146.53 (C, C-3), 146.49 (C, C-4), 141.29 (C, C-9), 131.05 (C, C-1), 129.69 (CH, C-7), 126.99 (CH, C-8), 120.18 (CH, C-6), 116.41 (CH, C-5), 113.81 (CH, C-2), 105.74 (2CH, C-10, C-14), 102.62 (CH, C-12). LC-ESI-MS (positive mode): *m/z* 245.1 $[M+H]^+$ (100); LC-ESI-MS (negative mode): *m/z* 243.1 $[M-H]^-$ (100), 478.1 $[2M-H]^-$ (18).

*trans-Scirpusin B (**17**)* [$C_{28}H_{22}O_8$]: Yellowish-brownish solid; $[\alpha]_D^{20}$ - 18.2 (MeOH; *c* 0.1); ^{1}H NMR (400.19 MHz, MeOH-d_4) δ_H (in ppm) 6.77 (1H, d, *J* = 16.2 Hz, H-7b), 6.76 (1H, d, *J* = 2.4 Hz, H-2a), 6.74 (1H, d, *J* = 8.3 Hz, H-5a), 6.70 (1H, d, J = 1.9 Hz, H-2b), 6.64 (2H, d, *J* = 8.2 Hz, H-5b, H-6a), 6.62 (1H, d, *J* = 2.1 Hz, H-14b), 6.58 (1H, dd,

J = 9.0, 2.7 Hz, H-6b), 6.54 (1H, d, *J* = 16.3 Hz, H-8b), 6.26 (1H, d, *J* = 2.0 Hz, H-12b), 6.18 (1H, t-like, H-12a), 6.16 (1H, s, H-10a), 6.15 (1H, s, H-14a), 5.28 (1H, d, *J* = 5.8 Hz, H-7a), 4.34 (1H, d, *J* = 5.8 Hz, H-8a). ^{13}C NMR (150.91 MHz, MeOH-d_4) δc (in ppm) 162.86 (C, C-11b), 159.97 (2C, C-11a, C-13a), 159.76 (C, C-13b), 147.66 (C, C-9a), 146.61*[1] (C, C-4b), 146.50*[1] (C, C-3b), 146.42*[2] (C, C-4a), 146.31*[2] (C, C-3a), 137.03 (C, C-9b), 134.96 (C, C-1a), 130.99*[3] (C, C-1b), 130.90*[3] (CH, C-7b), 123.65 (CH, C-8b), 120.03 (CH, C-6b), 119.84 (C, C-10b), 118.46 (CH, C-6a), 116.45 (CH, C-5b), 116.27 (CH, C-5a), 114.08 (CH, C-2b). 113.66 (CH, C-2a), 107.33 (2CH, C-10a, C-14a), 104.43 (CH, C-14b), 102.24 (CH, C-12a). 96.82 (CH, C-12b), 94.90 (CH, C-7a), 58.12 (CH, C-8a). *[1,2] The assignments of C-3b and C-4b, of C-3a and C-4a and of C-1b and C-7b may be exchangeable and are labeled with an asterisk. LC-ESI-MS (positive mode): *m/z* 487.1 $[M+H]^+$ (100); LC-ESI-MS (negative mode): *m/z* 485.1 $[M-H]^-$ (100), 971.1 $[2M-H]^-$ (70).

*trans-Sobrerol (**18**)* [$C_{10}H_{18}O_2$]: White solid; $[\alpha]_D^{20}$ - 4.4 ($CHCl_3$; *c* 0.1); ^{1}H NMR (400.19 MHz, MeOH-d_4) δ_H (in ppm) 5.57 (1H, d, *J* = 5.4 Hz, H-6), 3.98 (1H, dd, *J* = 4.0, 2.2 Hz, H-2), 2.12 (1H, m, H-5a), 1.97 (1H, dd, *J* = 13.9, 2.2 Hz, H-3a), 1.80 (1H, s, H-5b), 1.77 (4H, s, H-4, H-10), 1.37 (1H, m, H-3b), 1.18 (6H, s, H-8, H-9). ^{13}C NMR (150.91 MHz, MeOH-d_4) δc (in ppm) 135.41 (C, C-1), 126.17 (CH, C-6), 72.78 (C, C-7), 69.25 (CH, C-2), 39.76 (CH, C-4), 34.18 (CH_2, C-3), 28.04 (CH_2, C-5), 27.12 (CH_3, C-9), 26.94 (CH_3, C-8), 21.15 (CH_3, C-10). LC-ESI-MS (positive mode): *m/z* 193.1 $[M+Na]^+$ (10).

*Cyperusphenol B (**19**)* [$C_{42}H_{32}O_{12}$]: Brownish solid; $[\alpha]_D^{20}$ - 17.6 (MeOH; *c* 0.1); ^{1}H NMR (400.19 MHz, acetone-d_6) δ_H (in ppm) 6.84 (1H, d, *J* = 2.4 Hz, H-14a), 6.76 (1H, d, *J* = 1.8 Hz, H-2a), 6.74*[1] (1H, d, *J* = 2.1 Hz, H-2c), 6.72*[1] (1H, d, *J* = 8.1 Hz, H-5a), 6.69 (1H, dd, *J* = 8.3, 1.9 Hz, H-6a), 6.66 (1H, d, *J* = 8.1 Hz, H-5c), 6.56 (1H, dd, *J* = 8.2. 2.1 Hz, H-6c), 6.50 (1H, s, H-5b), 6.41 (1H, d, *J* = 2.3 Hz, H-12a), 6.39 (1H, d, *J* = 1.9 Hz, H-14b), 6.27 (1H, brs, H-7a), 6.22 (2H, d, *J* = 2.2 Hz, H-10c, H-14c), 6.12 (1H, t, *J* = 2.1 Hz, H-12c), 6.07 (1H, s, H-2b), 5.99 (1H, d, *J* = 1.9 Hz, H-12b), 4.65 (1H, d, *J* = 11.2 Hz, H-7b), 4.29 (1H, brs, H-8a), 4.01 (1H, d, *J* = 3.7 Hz, H-7c), 3.67 (1H, dd, *J* = 11.4, 3.8 Hz, H-8c), 3.56 (1H, dd, *J* = 12.0, 11.7 Hz, H-8b). ^{13}C NMR (150.91 MHz, acetone-d_6) δc (in ppm) 159.48 (C, C-13b), 159.34 (2C, C-11c, C-13c), 158.95 (C, C-11a), 157.90 (C, C-11b), 156.78 (C, C-13a), 151.38 (C, C-9c), 145.54*[2] (C, C-3a), 145.51*[2] (C, C-4a), 145.64*[2] (C, C-3c), 143.93*[2] (C, C-3b), 143.76*[2] (2C, C-4b, C-4c), 142.68 (C, C-9a), 140.70 (C, C-1c), 137.96 (C, C-1b), 137.76 (C, C-9b), 135.00 (C, C-1a), 130.98 (C, C-6b), 124.46 (C, C-10b), 120.03 (CH, C-6c), 117.62 (CH, C-6a), 117.14 (CH, C-5b), 116.49 (C, C-10a), 116.06 (2CH, C-5a, C-2c), 115.63 (CH, C-5c), 113.19 (CH, C-2a), 112.53 (CH, C-2b), 106.92 (CH, C-14b), 106.82 (2CH, C-10c, C-14c), 103.63 (CH, C-14a), 101.74 (CH, C-12a), 101.18 (CH, C-12c), 95.10 (CH, C-12b), 84.94 (CH, C-7a), 55. 53 (CH, C-7c), 55.02 (CH, C-8c), 52.06 (CH, C-8a), 51.20 (CH, C-8b), 43.16 (CH, C-7b). *[1,2] The assignments of H-5a and H-2c and of C-3a, C-4a, C-3b, C-4b, C-3c and C-4c may be exchangeable and are labeled with an asterisk. LC-ESI-MS (positive mode): *m/z* 729.2 $[M+H]^+$ (100); LC-ESI-MS (negative mode): *m/z* 727.2 $[M-H]^-$ (100).

Phytochemical and pharmacological analysis of the most active fractions

Figure S1. Cell viability of the obtained PE, DEE, PRC, EtOAc, 1-BuOH and H_2O fractions.

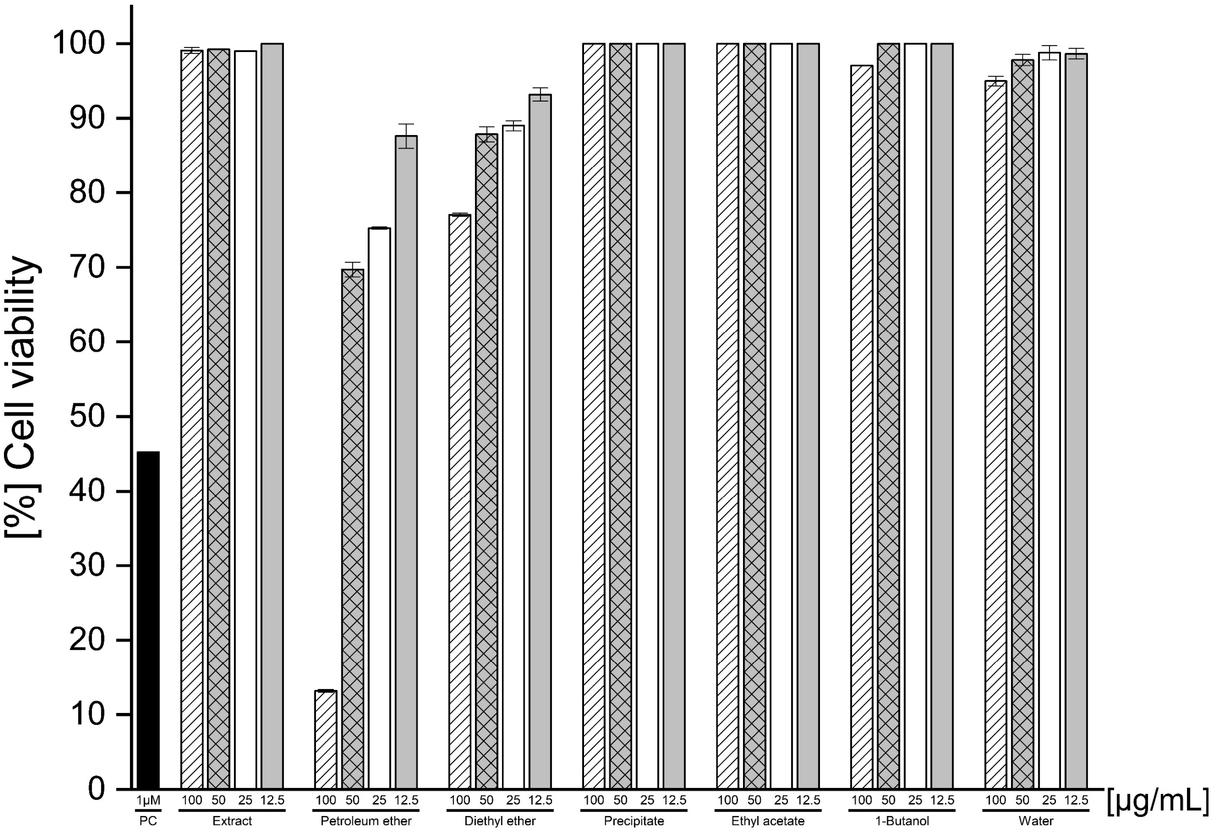

The MTT activity is expressed as relative percentage to that in the untreated group. LPS-stimulated J774A.1 murine macrophages were incubated with methanol extract and its subfractions (petroleum ether, diethyl ether, precipitate, ethyl acetate, 1-butanol and water) at concentrations of 100, 50, 25 and 12.5 µg mL^{-1}, followed by measurement of the cell viability vs LPS ± SEM (n=3). 6-MP was used as positive control (PC) at a concentration of 1 µM.

Figure S2. HPLC-UV chromatogram (205 nm) of DEE subfractions A–R after separation by *NP*-MPLC.

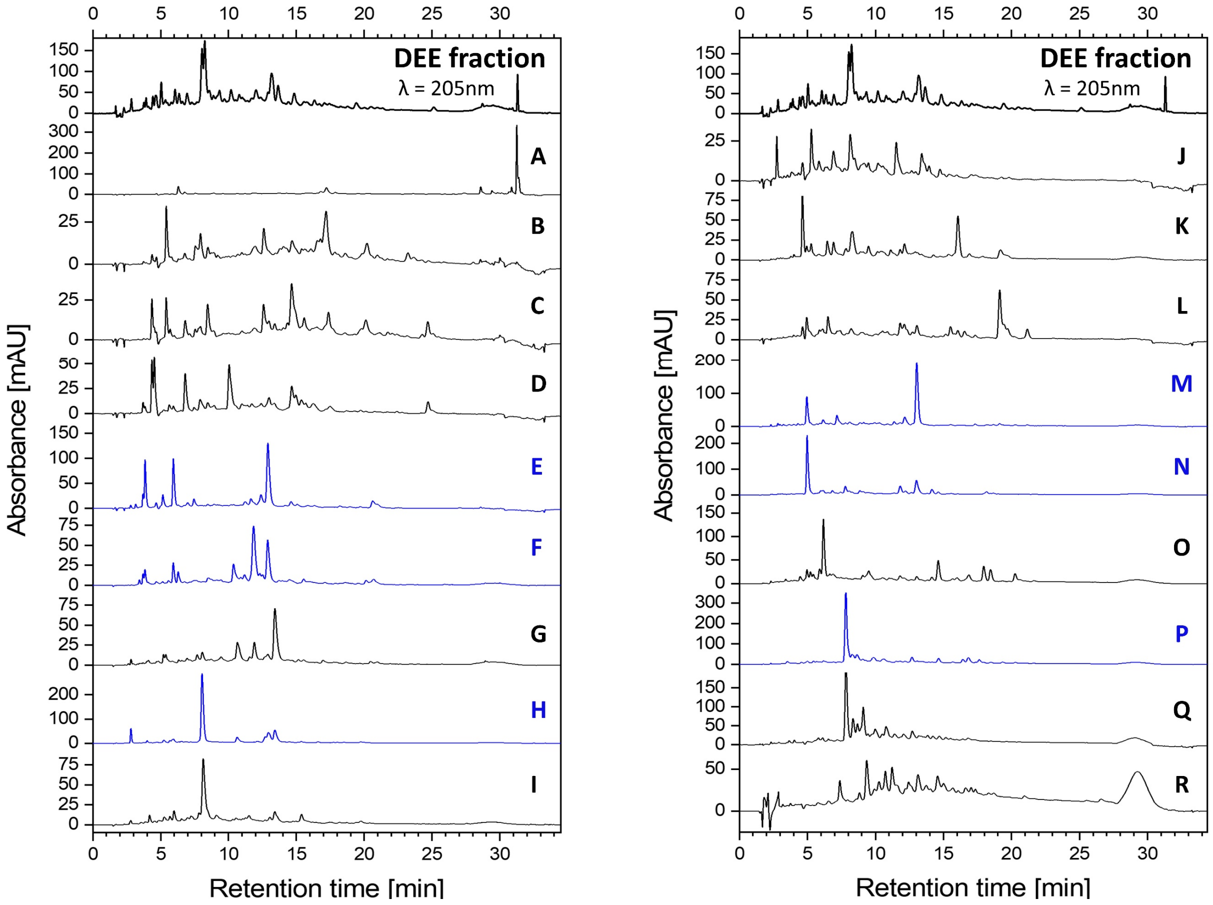

All fractions were tested for their pharmacological profile and fractions E, F, H, M, N, and P were selected for further examination.

Figure S3. Cell viability of DEE subfractions A–R of the methanolic extract of the subaerial parts of *C. articulatus*.

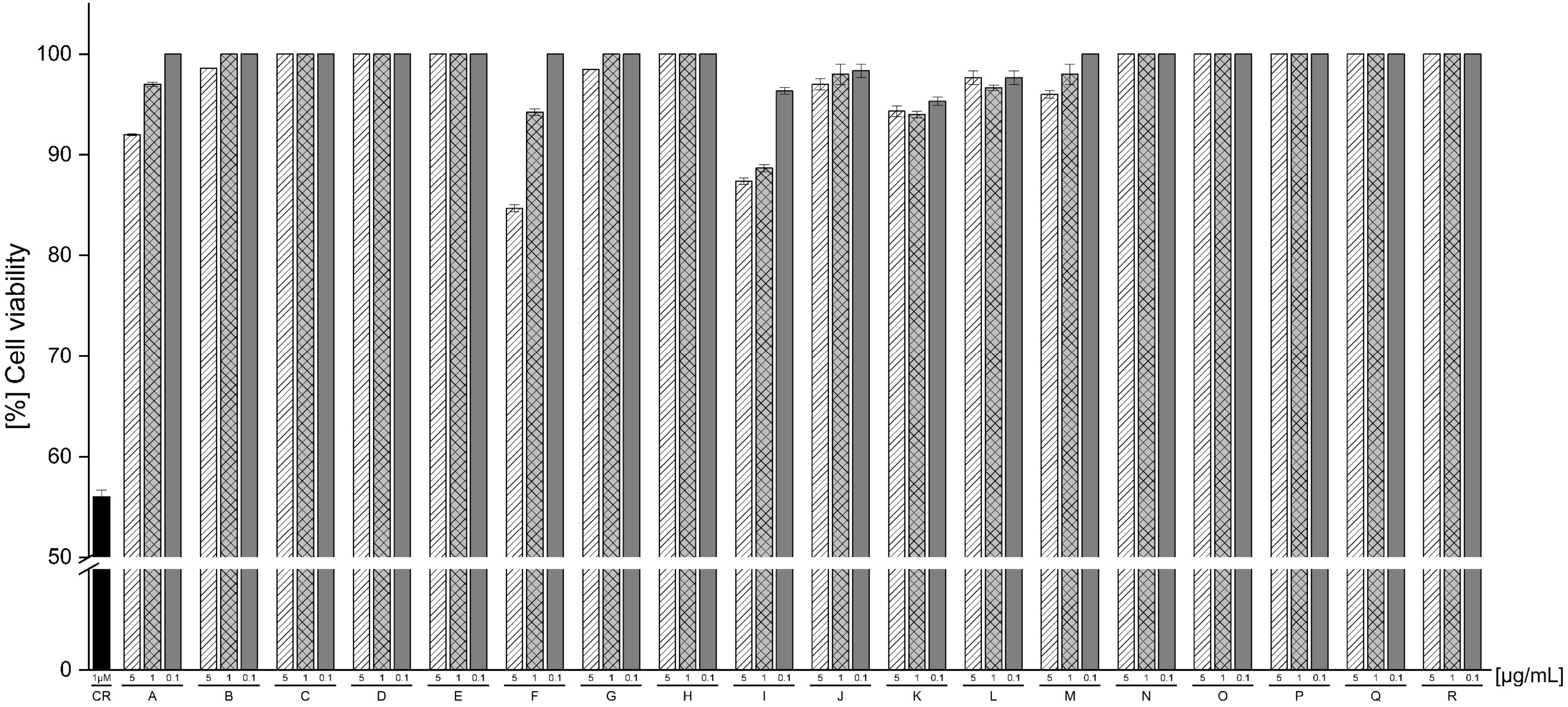

The MTT activity is expressed as relative percentage to that in the untreated group. LPS-stimulated J774A.1 murine macrophages were incubated with subfractions (A–R) at concentrations of 5, 1 and 0.1 µg mL^{-1}, followed by measurement of the cell viability vs LPS ± SEM (n=3). 6-MP was used as positive control (PC) at a concentration of 1 µM

Figure S4. GC chromatogram and peak assignment of the PE fraction of the methanolic extract of the subaerial parts of *C. articulatus*.

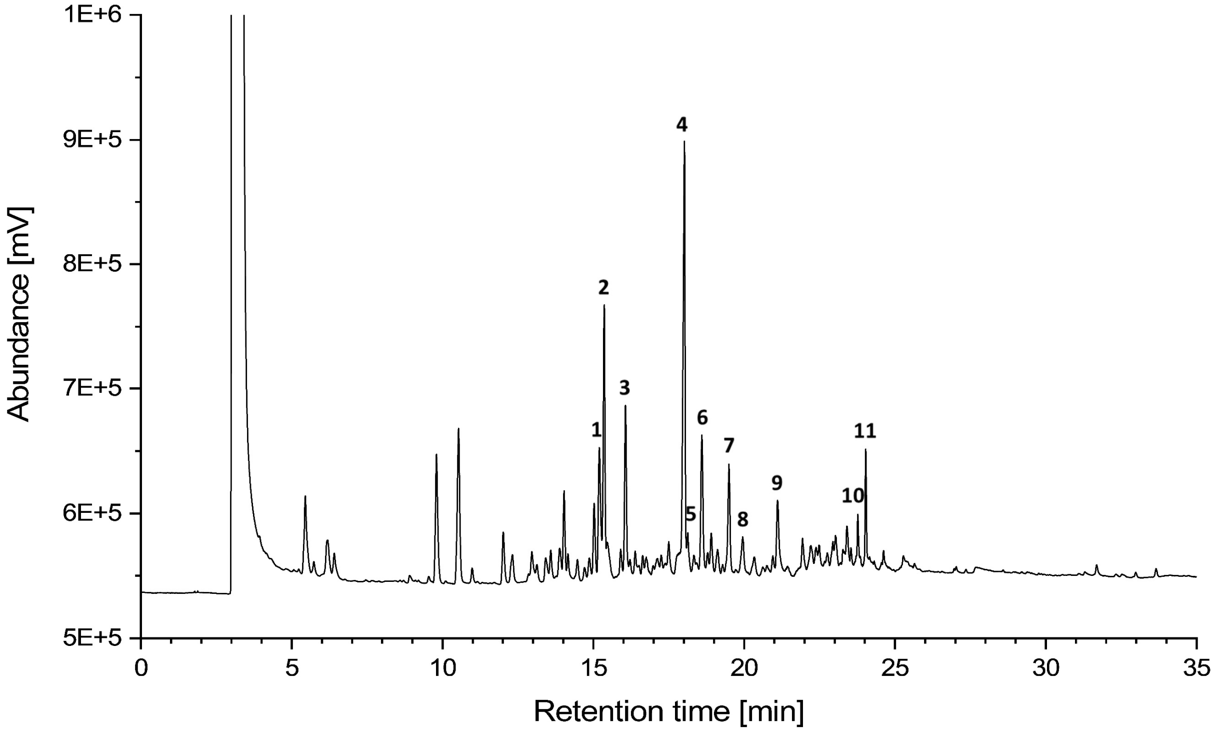

For GC conditions see **4.2** (General experimental methods).

Table S1. Antiproliferative activity and NO production inhibition of compounds **1**–**11** obtained from the PE fraction of the subaerial parts of *C. articulatus*.

		Antiproliferative activity (% activity ± SEM vs LPS)				NO production (% inhibition ± SEM vs LPS)		
**PE compound**		**20 µM**	**10 µM**	**5 µM**		**20 µM**	**10 µM**	**5 µM**
1		23.50 ± 3.50	8.00 ± 0.00	7.50 ± 2.50		35.00 ± 0.00	20.00 ± 2.00	11.50 ±0.50
2		0.00 ± 0.00	0.00 ± 0.00	0.00 ± 0.00		10.50 ± 0.50	8.50 ± 2.50	0.00 ± 0.00
3		12.50 ± 4.50	9.50 ± 0.50	0.50 ± 0.50		17.50 ± 0.50	17.50 ± 5.50	0.00 ± 0.00
4		17.50 ± 2.50	14.50 ± 2.50	5.50 ± 2.50		28.50 ± 0.50	27.00 ± 0.00	3.00 ± 0.00
5		17.50 ± 1.50	12.50 ± 0.00	0.50 ± 2.50		32.00 ± 0.00	30.50 ± 0.50	6.50 ± 0.50
6		3.50 ± 0.30	1.50 ± 0.10	0.50 ± 0.50		18.50 ± 2.50	16.00 ± 2.00	3.00 ±3.00
7		4.50 ± 0.40	1.00 ± 0.00	0.00 ±0.00		31.50 ± 3.50	22.00 ± 0.00	3.00 ± 3.00
9		13.50 ± 0.60	11.50 ± 0.50	2.50 ± 0.50		32.00 ± 1.00	25.00 ± 1.00	8.50 ± 1.50
10		16.00 ± 0.20	14.50 ± 0.05	0.50 ± 0.73		21.50 ± 4.50	11.50 ± 6.50	6.50 ± 0.50
11		0.00 ± 0.00	0.00 ± 0.00	0.00 ± 0.00		21.50 ± 0.50	13.50 ± 0.50	14.00 ± 0.00
**positive control**		**1 µM**				**1 µM**		
6-MP		47.33 ± 1.85						
L-NAME						44.00 ± 0.50		

Inhibition assays vs LPS ± SEM (n=3) at concentrations of 20, 10 and 5 µM.

Table S2. Antiproliferative activity of compounds 12–19 obtained from the DEE fraction of the subaerial parts of *C. articulatus*.

		**Antiproliferative activity (% activity ± SEM vs LPS)**		
**DEE compound**		**10 µM**	**5 µM**	**1 µM**
12		2.83 ± 0.33	0.00 ± 0.00	0.00 ± 0.00
13		18.50 ± 0.50	10.33 ± 0.33	7.67 ± 0.67
14		0.00 ± 0.00	0.00 ± 0.00	0.00 ± 0.00
15		0.00 ± 0.00	0.00 ± 0.00	0.00 ± 0.00
16		0.00 ± 0.00	0.00 ± 0.00	0.00 ± 0.00
17		2.50 ± 0.01	1.90 ± 0.33	0.00 ± 0.00
18		35.00 ± 0.01	24.00 ± 0.56	12.00 ±0.10
19		3.00 ± 0.33	2.67 ± 0.67	2.33 ± 0.33
**positive control**		**1 µM**		
6-MP		46.54 ± 0.35		
L-NAME				

Inhibition assays vs LPS ± SEM (n=3) at concentrations of 10, 5 and 1 µM.

Figure S5. Comparison of the chromatograms (205 nm) of the HPLC-UV analysis of extract subfractions of *S. holoschoenus* and *C. articulatus*.

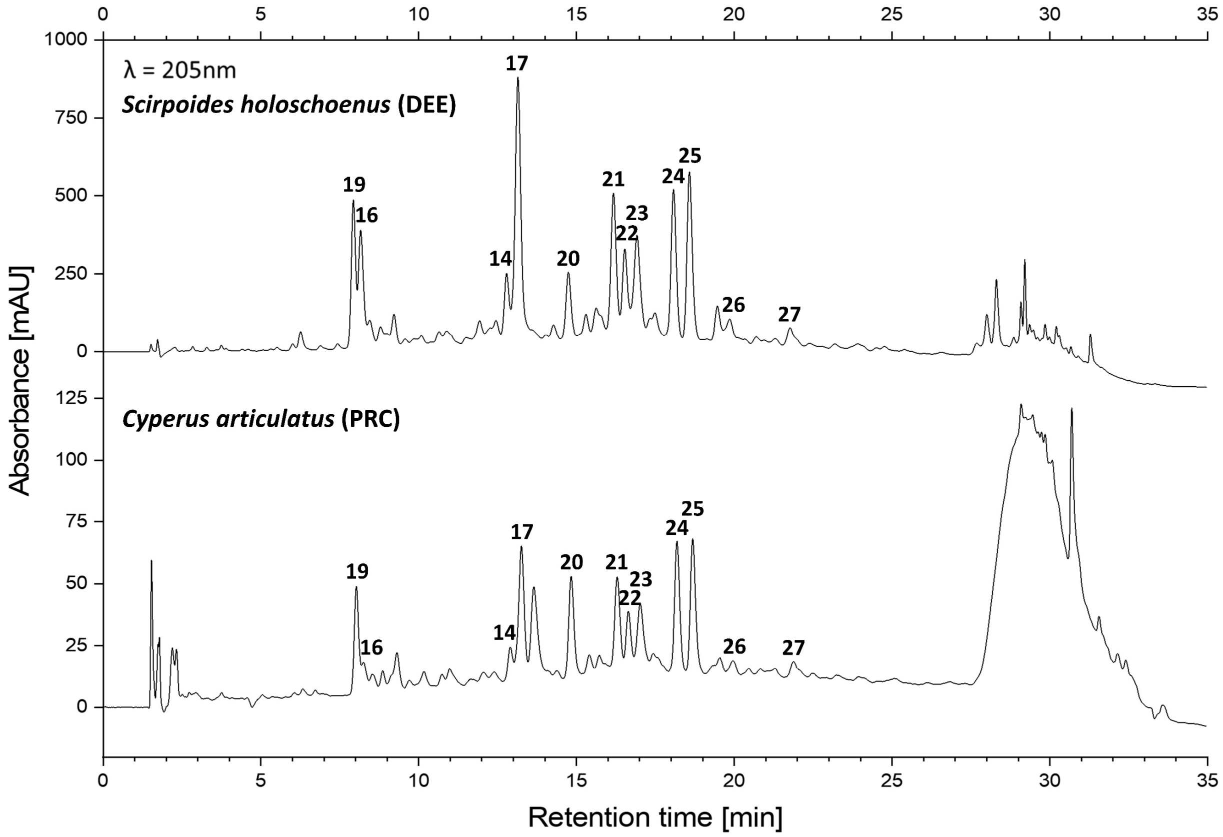

HPLC-UV chromatogram (205 nm) of the DEE fraction of the methanolic rhizomes and roots extract after DCM extraction of *S. holoschoenus* vs. the PRC fraction of the methanolic rhizome and root extract of *C. articulatus*.
